# Monitoring Brain Tumor Vascular Heamodynamic following Anti-Angiogenic Therapy with Advanced Magnetic Resonance Imaging in Mice

**DOI:** 10.1371/journal.pone.0115093

**Published:** 2014-12-15

**Authors:** Shlomi Laufer, Ahinoam Mazuz, Nathalie Nachmansson, Yakov Fellig, Benjamin William Corn, Felix Bokstein, Dafna Ben Bashat, Rinat Abramovitch

**Affiliations:** 1 The Goldyne Savad Institute for Gene Therapy, Hadassah Hebrew University Medical Center, Jerusalem, Israel; 2 MRI/MRS lab HBRC, Hadassah Hebrew University Medical Center, Jerusalem, Israel; 3 Pathology, Hadassah Hebrew University Medical Center, Jerusalem, Israel; 4 Institute of Radiotherapy, Tel Aviv Sourasky Medical Center, Tel Aviv, Israel; 5 Neuro-Oncology Service. Tel Aviv Sourasky Medical Center, Tel Aviv, Israel; 6 The Functional Brain Center, The Wohl Institute for Advanced Imaging, Tel Aviv Sourasky Medical Center, Tel Aviv, Israel; 7 Sackler Faculty of Medicine, Tel Aviv University, Tel Aviv, Israel; 8 Sagol School of Neuroscience, Tel Aviv University, Tel Aviv, Israel; University Hospital of Navarra, Spain

## Abstract

Advanced MR imaging methods have an essential role in classification, grading, follow-up and therapeutic management in patients with brain tumors. With the introduction of new therapeutic options, the challenge for better tissue characterization and diagnosis increase, calling for new reliable non-invasive imaging methods. In the current study we evaluated the added value of a combined protocol of blood oxygen level dependent (BOLD) imaging during hyperoxic challenge (termed hemodynamic response imaging (HRI)) in an orthotopic mouse model for glioblastoma under anti-angiogenic treatment with B20-4.1.1, an anti-VEGF antibody. In glioblastoma tumors, the elevated HRI indicated progressive angiogenesis as further confirmed by histology. In the current glioblastoma model, B20-treatment caused delayed tumor progression with no significant changes in HRI yet with slightly reduced tumor vascularity as indicated by histology. Furthermore, fewer apoptotic cells and higher proliferation index were detected in the B20-treated tumors compared to control-treated tumors. In conclusion, HRI provides an easy, safe and contrast agent free method for the assessment of the brain hemodynamic function, an additionally important clinical information.

## Introduction

Glioblastoma is the most common primary malignant brain tumor with two-year survival rates of less than 30%. Despite aggressive surgery, radiation therapy, and chemotherapy, median survival remains in the range of 15 months. The hallmarks of glioblastoma include rapid progression and high degree of vascularity [1], [2]. Several therapeutic approaches have been tested to treat glioblastoma tumors, but none of these can extend survival for more than a few months. In recent years, significant research efforts have focused on the use of anti-angiogenic therapies for the treatment of glioblastoma. These drugs have the potential to normalize abnormal tumor vasculature structurally and functionally, reduce the risk of hemorrhage, enhance the penetration of concurrently administered chemotherapy and improve the efficacy of cytotoxic drugs and radiation by alleviating hypoxia [3], [4]. Bevacizumab (Avastin), a monoclonal antibody that inactivates vascular endothelial growth factor (VEGF), was lately approved by the US Food and Drug Administration for treatment of recurrent glioblastoma. It reduces MRI enhancement, and provides benefit by controlling peritumoral edema and improving clinical performance. Its clinical use is becoming more widespread, even though its effect on overall survival and its anti-glioma effect remain questionable. Besides angiogenesis [1], phenomena such as vascular co-option and vascular mimicry were also evident in glioblastoma, especially following anti-angiogenic therapies [5].

Magnetic resonance images (MRI) is the method-of-choice for noninvasive whole brain assessment of brain tumors, having an essential role in classification, grading, follow-up and therapeutic management, due to its soft tissue resolution, safety and diversity. MRI can provide structural, biochemical and functional information regarding the tumor and its surrounding parenchyma. From previous studies, it has become clear that the conventional assessment of radiation effects and especially the efficacy of anti-tumor drugs by measuring the enhanced tumor area alone may not be the most appropriate endpoint. The successful introduction of anti-angiogenic therapies into clinical trials requires the development of reliable non-invasive methods for assessing angiogenesis and its modulation or inhibition in-vivo. Thus, in the last few years, a broad range of MRI techniques have been developed to provide feedback and surrogate markers for therapeutic response including tumor blood volume, perfusion, vessel permeability, oxygenation and vessel size [6], [7]. These methods, aimed at the early detection of vascular changes in response to therapy, may guide patient management based on the individual response pattern. Contrast enhanced (CE)- MRI is widely established and currently is the preferred method for brain tumor assessment. However, CE-MRI does not adequately assess disease status especially during Bevacizumab therapy for recurrent glioblastoma since recurrence is commonly associated with non-enhancement on CE-MRI [8]–[10].

Blood oxygenation level-dependent (BOLD) MRI uses the paramagnetic nature of deoxygenated hemoglobin versus the diamagnetic nature oxygenated hemoglobin [11]. Using this method, hemoglobin can serve as an endogenous contrast agent which indirectly represents changes in blood flow, volume and oxygenation. BOLD MRI is the basis for the well-established functional MRI (fMRI) method [12], in which hemodynamic changes due to neuronal activation are monitored. Changes in BOLD signal can also occur due to respiratory challenges of hyperoxia or hypercapnia. Pure oxygen inhalation causes increased blood oxygenation and reduced blood flow [13], while inhalation of a mixture of oxygen and CO_2_ (i.e. carbogen) has been shown to increased blood oxygenation and flow. Typically, when respiratory challenges are viewed, a T_2_* sequence is used [14], [15]. Previously, we utilized Hemodynamic Response Imaging (HRI), an fMRI method combined with hypercapnic and hyperoxic challenges for functional analysis of vessel maturation (which can render resistance to anti-angiogenic therapy), and vessel density and functionality [16]. Using changes in the BOLD signal induced by respiratory challenges is not limited to brain imaging and can be used as a tool for assessing the hemodynamic response in different organs and pathologies. Several animal studies were performed to estimate treatment response in liver metastases [17]; classify liver fibrosis [18]; evaluate renal perfusion and hemodynamics during acute kidney injury [19]; and study tumor vasculature [20]. Also, several studies utilized rodent brain models in order to distinguish neural from non-neural contributions to fMRI signals [21] and evaluated BOLD signal changes due to hyperoxia and hypercapnia [22].

Recently, our group reported a clinical study demonstrating the use of HRI for the assessment of angiogenesis in brain tumors both in untreated tumors and in tumors treated with chemotherapy and radiation therapy [23]. The aim of the current study focused on the development of equivalent mouse model, which will enable us to perform more fundamental research in order to achieve better understanding of the involved cellular processes and to elucidate the imaging characteristics. In this context an orthotopic mouse model for brain glioblastoma tumors was established. In this model, the effect of an anti-angiogenic treatment with B20-4.1.1 (B20) was evaluated. B20 is an anti-VEGF antibody which has previously been used as a surrogate for preclinical modeling of bevacizumab activity, since it has affinity toward VEGF similar to bevacizumab, yet effectively blocks both the human and murine ligands [24], [25]. The MRI results were further confirmed by histological assessment.

## Materials and Methods

### Materials

B20-4.1.1 (B20), the human- and mouse-VEGF-binding monoclonal antibody [24], [25], was provided from Genentech Inc., (San Francisco, CA).

### Animal studies

Animal experiments were performed in accordance with the guidelines and approval of the Animal Care and Use Committee of the Hebrew University, which holds the NIH approval (OPRR-A01-5011). Male CB6F1 mice (7–8 weeks old, Harlan Laboratories, Jerusalem, Israel) were used and received humane care from a properly trained professional. All animals were kept in a temperature and light controlled room with a 12-hr light 12-hr darkness cycle, and received food and water ad libitum.

#### Primary brain glioma model

Gl-261 murine glioma cells [26] were cultured in DMEM supplemented with 10% FCS (Biological Industries Israel) and 1% penicillin, streptomycin and L- Glutamate (Biological Industries Israel). A total of 21 mice were included in this study. On the day of the procedure, the mice were anesthetized using a ketamine/xylasine combination (160 mg kg^−1^ and 6.5 mg kg^−1^, respectively) and Gl-261 glioma cells (10^5^ cells, 2 µl) were stereotactically injected using a Hamilton syringe (21 G) into the right hemisphere (0.6 mm anterior, 2.3 mm lateral to bregma at 3 mm depth from the dura). Treatment with B20 started at day 7 (n = 6) at a dose of 5 mg/kg that was given via intraperitoneal (IP) injection, three times per week. Animals were scanned on average, 3 times post tumor injection at days 7–8, 14–16 and 20–23.

### MRI

MRI scans were performed in a 4.7T Bruker Biospec spectrometer using a passively RF decoupled surface coil, 1.2-cm in diameter (Dotty Scientific Inc., Columbia, South Carolina), and a birdcage transmission coil (Bruker Corp. Germany). During the scan the mice were anesthetized using isoflurane (2%). In all MRI scans the slice thickness was 1 mm, field of view (FOV) was 18×18 mm^2^ and the matrix size was 128×128 (resulting with a voxel size of 1×0.14×0.14 mm^3^). The full MRI protocol included the following anatomical and advanced MR methods.

#### Anatomical MRI

True-FISP images in the horizontal and axial planes were acquired (true fast imaging with steady-state precession; time to repetition [TR] = 3 ms; time to echo [TE] = 1.5 ms). In addition T_2_-weighted (T_2_W) fast spin-echo images were obtained (TR = 3000 ms; TE = 55.4 ms) in the horizontal plane.

#### Diffusion Weighted Imaging (DWI)

Assessment of tumor cellular organization was achieved using DWI. In the brain tumor study the data were collected using a standard spin-echo pulse sequence (TR = 1500 ms; TE = 50 ms) with the addition of diffusion-sensitizing gradients with four b-values ranging from 0 to 1,600 s/mm^2^ with time between diffusion gradient pulses [D] = 50 ms and diffusion gradient duration [d] = 5 ms.

#### Hemodynamic Response Imaging (HRI)

Images were acquired using a T_2_*-weighted gradient echo sequence (TR = 155.9 ms; TE = 10 ms; 20 s per image). Map calculation method has been described previously [27], [28]. Briefly, images were acquired during breathing of air, and carbogen (95% oxygen and 5% CO_2_) alternately. Maps of mean signal intensity (SI) value for each pixel during the different inhaled gases (*S_air_* and *S_O2_*) were calculated. The percentage change of SI induced by hyperoxia, Δ*S_O2_*, was calculated for each pixel:
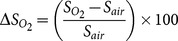



The MR SI was correlated to the breathing paradigms (block design analysis), and mean ΔS values for each region of interest (ROI) was calculated only from voxels that passed the statistical threshold of p<0.05 (active pixels).

In addition to the mean ΔS value, a graph of the average SI change over time was also calculated for each ROI. Two methods for calculating this average were used: In the first- only active pixels were included by averaging their percentage change compared to baseline; while, in the second method this average was further multiplied by the ratio of the number of active pixels divided by the total number of pixels in the ROI. Throughout this manuscript, the first method will be referred as time-course (TC) while the second will be referred as ROI time-course (RTC). In a more formal way:
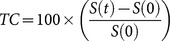


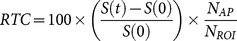



Where *S*(t) is the average SI of active pixels at time *t. S*(0) is the average SI at baseline (typically the first 4 repetitions, providing better stability than just the first repetition). *N_AP_* is the number of active pixels and *N_ROI_* is the total number of pixels in the given ROI.

### Data processing and analysis

The brain and tumor borders were segmented manually using ANALYZE software (Biomedical-Imaging Software System, Mayo Clinic) based on the true-FISP horizontal scans. The tumor contra lateral side (CLS) was defined by flipping the tumor ROI (left-right flip). The post-processing of the MR data from the advanced MR methods (HRI, DWI) and all other analysis was done using an in-house program written in Matlab (The MathWorks R2011a).

### Histological analysis

Anesthetized animals (ketamine/xylasine) were perfused transcardially with 10 ml of formalin. Brains were removed and immersed in the same fixative overnight. Formalin-fixed paraffin-embedded coronal sections (5 µm) were stained with hematoxylin and eosin (H&E) or subjected to immunohistochemistry with specific antibodies. For apoptosis assessment, TUNEL (Terminal deoxynucleotidyl transferase-mediated deoxyuridine triphosphate nick end-labeling) staining was performed using the in-situ cell death detection Kit (Roche Diagnostics Corp. Indianapolis, Ind), according to the manufacturer protocol. Proliferation estimation was performed by staining with monoclonal anti-Ki67 (SP6) antibody (1∶100, Neomarker). Tumor blood vessels were detected using anti-PECAM-1 antibody (CD31; 1∶50; Biocare Medical, Concord, California). Blood vessel maturation was detected using α-smooth muscle actin (α-SMA) antibody (1∶300; Sigma Chemical Co, St. Louis, MO). Diagnosis and other pathological interpretations were conducted by a senior pathologist. Immunostaining was evaluated in 10 randomly high-power microscopic fields (HPF) selected only in viable tumor regions (magnification X400), and the mean value of positive cells or vessels was calculated.

### Statistics

Statistical analyses were performed using Instat Biostatistics software (GraphPad Software Inc. San Diego, California). The Kolmogorov–Smirnov test was used for testing normal distributions of the data. The paired two-tailed t-test analysis was used to determine statistical significance between the tumor-border to tumor-center and between the tumor to CLS (for CD31, HRI and diffusion). The unpaired two-tailed t-test analysis was used to determine statistical significance between tumors treated with B20 and control treated tumors (for CD31, HRI and diffusion). We considered a two-tailed value of *p*<0.05 statistically significant. Results are expressed as means ± SD.

## Results

### Brain HRI optimization

**Figure 1:**
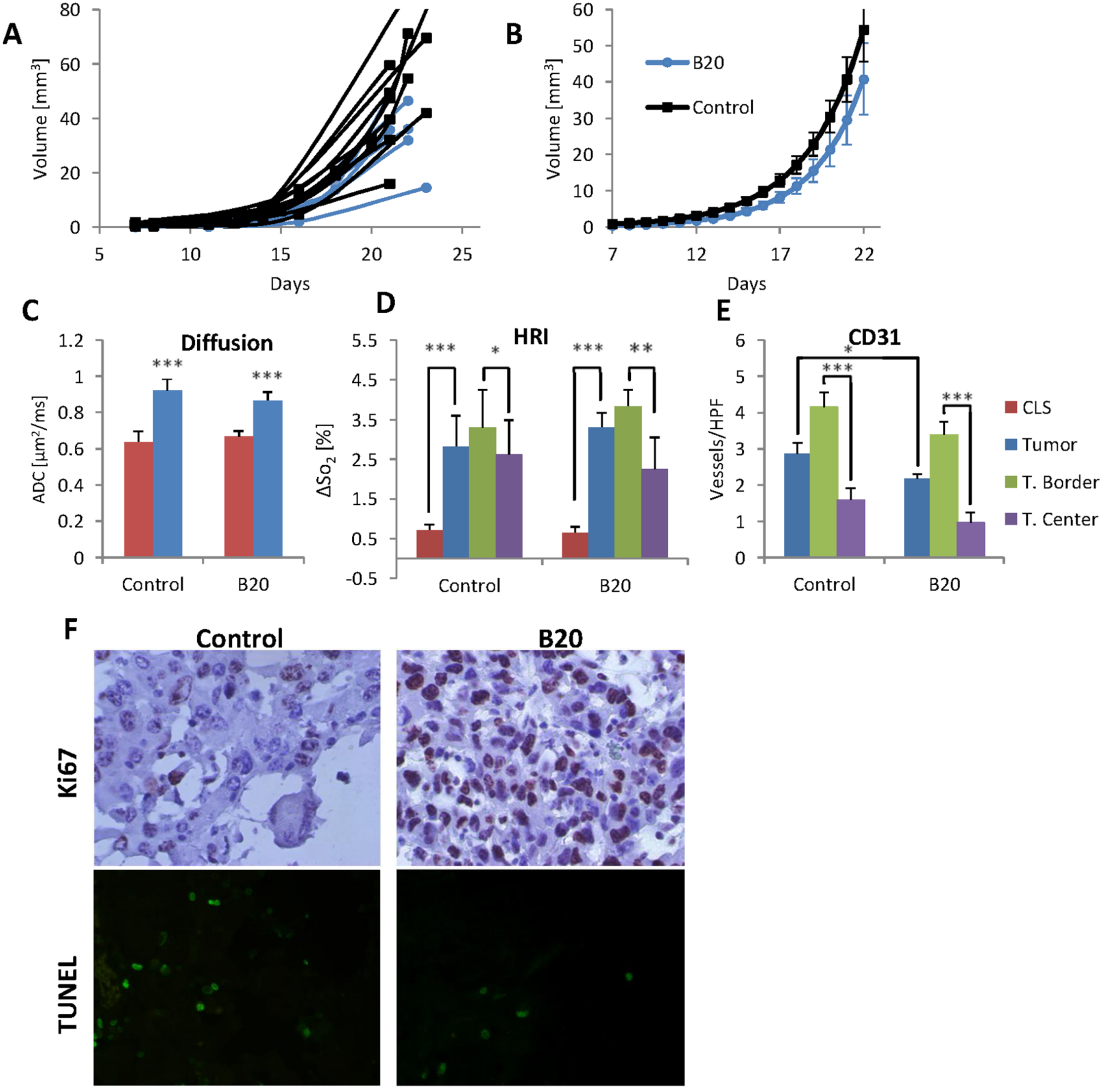
HRI in the healthy brain. HRI maps were obtained without (**A, B, C, E, F, G**) or with (**I, J, K, M, N, O**) constant air flow above the head. HRI maps are presented for both positive and negative pixels (**A, I**) and the corresponding SI time course (**E, M**); only for positive pixels (**B, J**) and the corresponding SI time course (**F, N**) and only for negative pixels (**C, K**) and the corresponding SI time course (**G, O**). In the graphs the time-curse (TC, red line) is calculated only for active pixels in the brain ROI (marked in blue on the HRI maps) and the ROI time-course (RTC, blue line) is the TC multiplied by the ratio of the number of active pixels divided by the total number of pixels in the ROI. It can be seen that when airflow is used the negative response is significantly lower and randomly distributed (**K**) compared to maps acquired without airflow (**C**). The corresponding T_2_W image is presented in (**D**) with the matching HRI superimposed on top (**H**). Note that the main blood vessels are clearly manifested on the HRI map. (**L**) The mean TC and RTC calculated from 44 HRI maps acquired with or without constant airflow are compared.

When performing brain-HRI in mice, numerous pixels with negative ΔS values were always detected in the upper slice in the vicinity of the skull, causing reduced total reactivity in the ROI (see example in **Fig.** 1A**–**C). In order to overcome this unexpected phenomenon, a constant air flow was introduced above the head, throughout the entire HRI sequence. The flow was delivered to the area of the surface coil and prevented negative BOLD in the upper slice, as can be seen in the example shown in **Fig.** 1. Notably, the prominent negative effect that was detected when no airflow was provided (**Fig.** 1A**–**C) was diminished when a constant airflow was delivered (**Fig.** 1I**–**K). In **Fig.** 1L the average time courses calculated from 44 naïve HRI maps performed with and without airflow are depicted. While the RTC is significantly reduced when no airflow is supplied, the TC is roughly the same.

### Monitoring tumor progression

**Figure 2:**
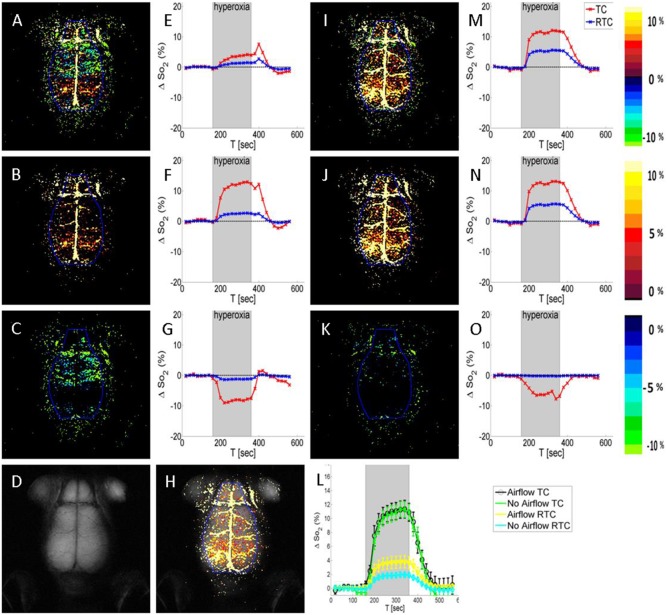
Glioblastoma tumor imaging characteristics. Representative True-FISP image (**B**), T_2_W image (**C**), DWI map (**D**) and HRI map (**E**) with the corresponding H&E stained histological slide (**A**) of control tumor obtained 23 days post Gl-261 murine glioma cells inoculation. The tumor is clearly identified on the True-FISP image with bright areas at the borders. These areas displayed high mean diffusivity values and matched vacant areas in the H&E slide (marked with arrows). Increased tumor vascularity at the periphery was evident on the HRI map which was further confirmed by histological evaluation. (**F**) Representative histological slide immunostained with the endothelial cell marker CD31 of control tumor. Photographs were taken from the peripheral (left) and central (right) regions (original magnification X40) demonstrating the higher vascularity in tumor periphery.

**Figure 3:**
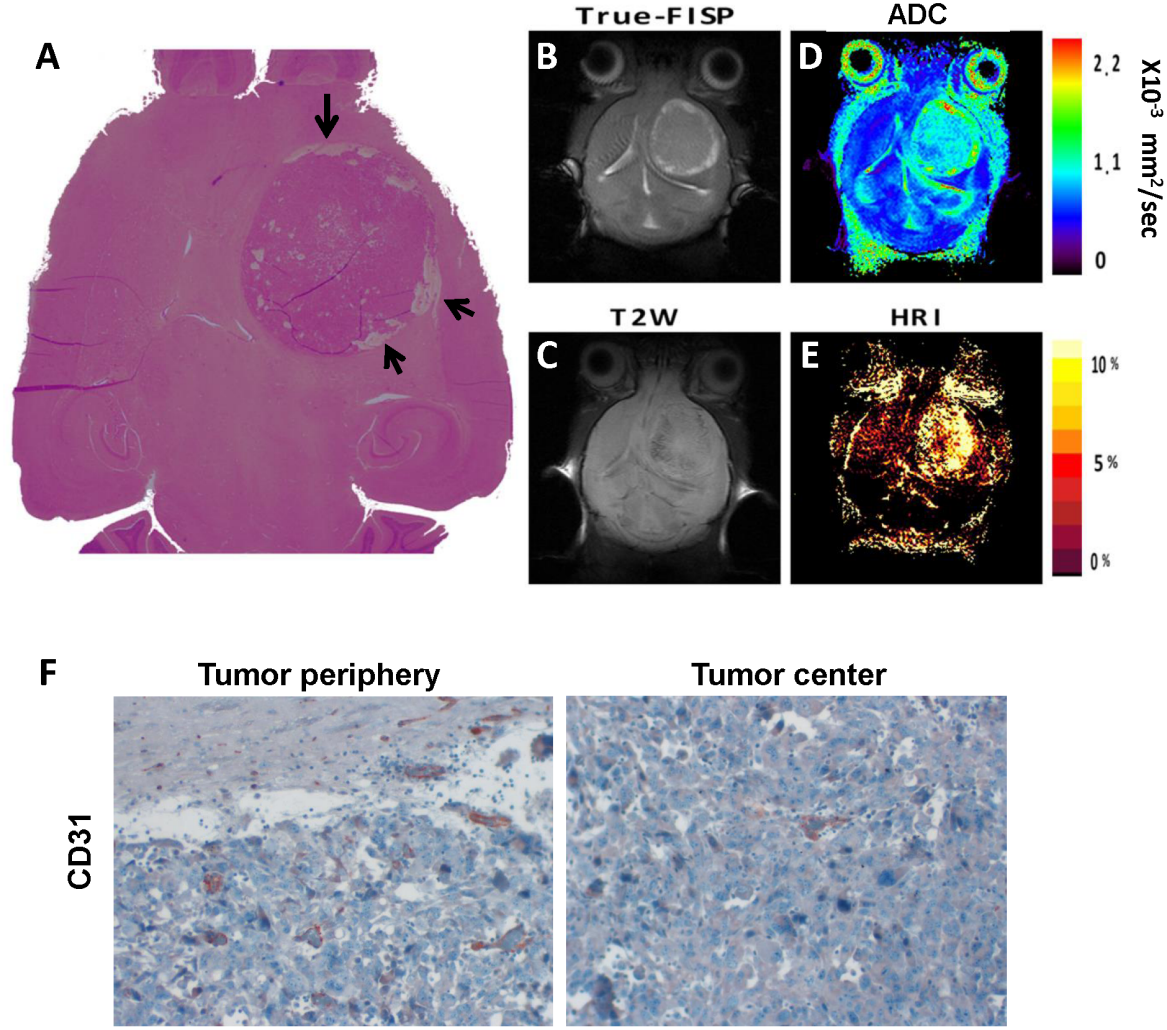
Glioblastoma tumor imaging characteristics. Representative T_2_W image (**A**), T_1_W image obtained before Gd-DTPA injection (**B**); contrast enhanced T_1_W image obtained 1 min after Gd-DTPA injection (**C**); DWI map (**D**) and HRI map (**E**) with the corresponding H&E stained histological slide (**F**) of control tumor obtained 30 days post Gl-261 murine glioma cells inoculation. T-tumor, E-edema; bar-1 cm.

**Figure 4:**
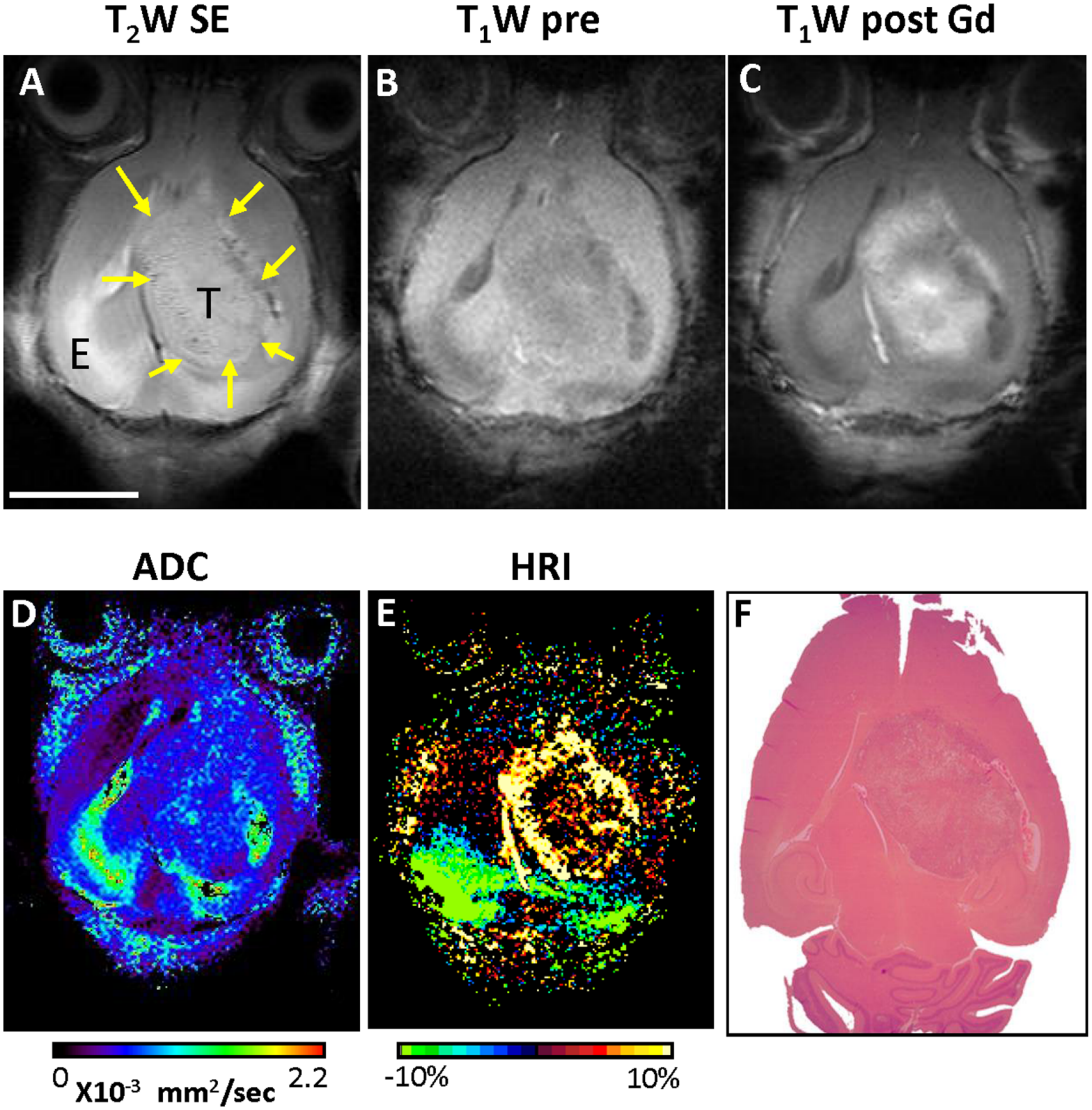
Glioblastoma imaging parameters progression over time. (**A**) Serial coronal FISP (first row) and T_2_W (second row) images of a control tumor that were acquired on days 7, 14, 18 and 22. The corresponding DWI (third row) and HRI (fourth row) maps are also shown. ROIs delineating the tumor and contra-lateral healthy brain tissue are marked on the FISP and T_2_W images (blue line). Increased HRI response during tumor progression can be seen both in the HRI maps and in the ROI-time-course (RTC) graphs (fifth row). The average diffusion values (**B**) and the HRI values (**C**) of the tumors (blue) and the contra-lateral side (CLS; red) are given as a function of time from cell inoculation. Note that the diffusion values are higher than the CLS throughout the entire period while tumor HRI increased over time. ***denotes statistical significance at P<0.005 compared to the CLS value.

The initial brain tumors detection was achieved using true-FISP images already 7 days post cell inoculation and tumor volume was easily measurable. By using several anatomical and functional MR sequences the heterogeneity inside these tumors was demonstrated. Two examples of the different MRI images acquired on day 23 (Fig. 2) or day 30 (Fig. 3) post tumor implantation, with the corresponding H&E histological slides. In the true-FISP images, the tumors appeared bright while in T_2_W images tumor borders were less distinct (Figs. 2–4). In addition, most of the tumors exhibited higher apparent diffusion coefficients (ADC) (Figs. 2, 4) and a higher hemodynamic response in HRI maps compared to the normal brain values (Fig. 2–4). In a subset of the tumors, higher ADC values were observed at the tumor border matching the bright areas in the true-FISP images and were further confirmed as hollow spaces detected on the H&E slide (marked with arrows on Fig. 2A). The darker areas in the T_2_W images represent areas of increased vascular density at tumor periphery as confirmed with HRI (Figs. 2–3).

**Figure 5:**
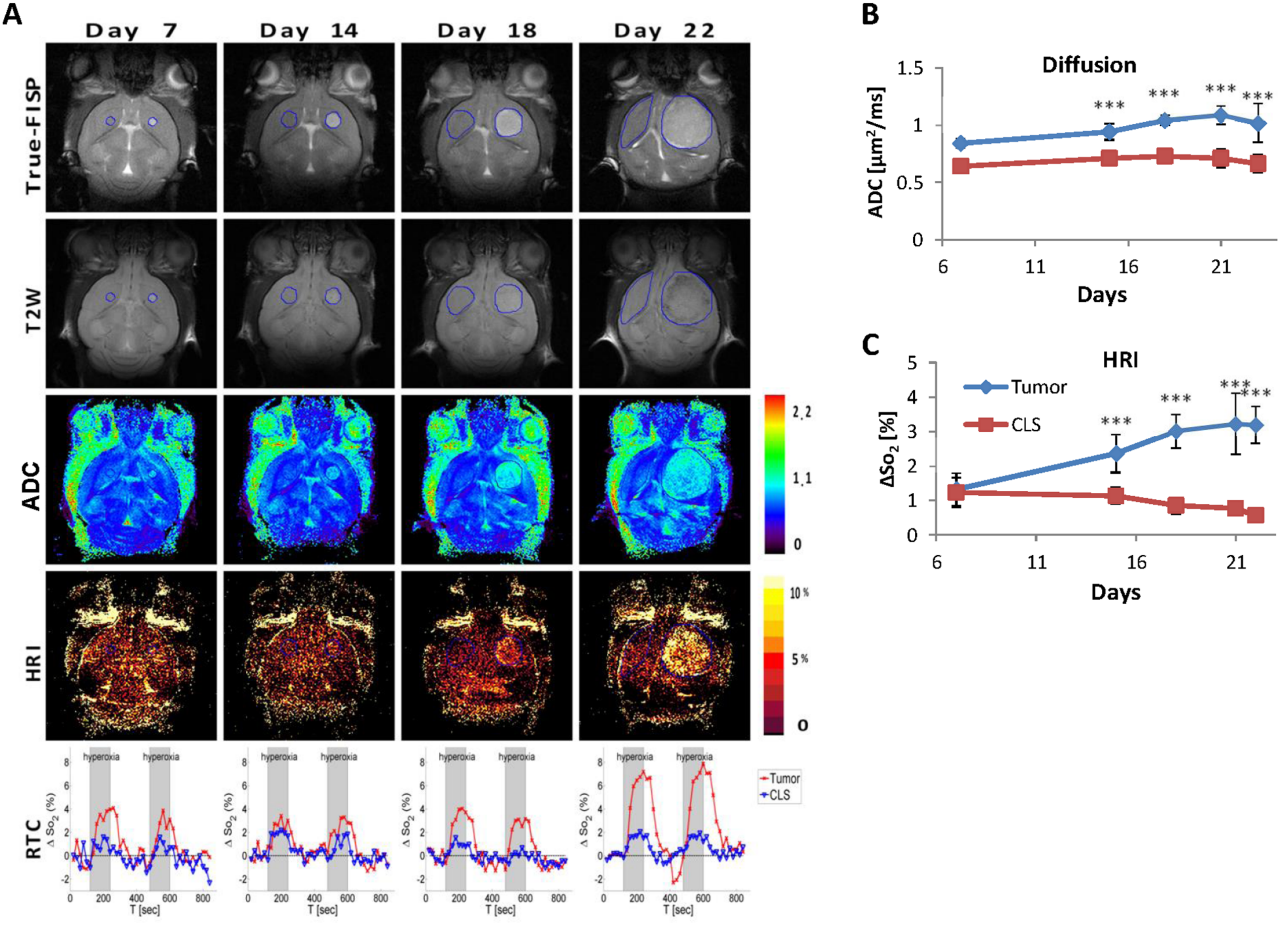
Effects of B20 therapy on glioblastoma tumors. Tumor volume (mm^3^) for each individual tumor (**A**) and the average volume of the entire group (**B**), as a function of days post cell inoculation for control (black, n = 15) and B20 treated (blue, n = 6) mice. B20 showed only moderate effects on tumor growth kinetics. Comparison of the average diffusion (**C**) and HRI (**D**) values of tumors (blue; larger than 15 mm^3^) and the CLS (red) between control and B20 treated mice. Additionally, the HRI tumor values were also separated between borders (green) and the central area (purple). (**E**) Quantification of vessel count/HPF analyzed from 10 randomly selected HPF/tumor is based on the CD31 immunostaining. Tumor vascularity is significantly higher in the border (green) compared to the center (purple) in both groups. (**F**) Representative histological sections of control (left) and B20 treated (right) immunostained with Ki67 for proliferation (**top**) and TUNEL for apoptosis (**bottom**); Original magnification X40. *denotes statistical significance at P<0.05; **denotes statistical significance at P<0.01; ***denotes statistical significance at P<0.005.

Tumor growth kinetics monitoring was assessed by longitudinal MRI scans (Figs. 4A and 5A). The progression of a representative control glioblastoma tumor over time can be seen in Fig. 4A. On true-FISP images (first row) the tumors appeared brighter compared to the adjacent brain tissue throughout the entire progression. Furthermore, higher diffusion values are measured in the tumor during the entire progress period (third row). Mean ADC were significantly higher in the tumors in compression to the CLS (Fig. 3B). This difference was observed already at day 7 with diffusion values of 0.84 and 0.64 µm^2^/sec in tumor and CLS, respectively. There was a moderate increase in tumor diffusion to 1.02 compared to 0.66 in CLS towards the end (day 21).

In the corresponding HRI maps (forth row) a low response was evident in the tumor at the early growth phase (up to two weeks) with increased response in the advanced growth phase of the tumor (Fig. 4C). The tumors and the CLS had similar HRI values at day 7 with mean values of 1.3% and 1.23% in the tumor and in the CLS, respectively (Fig. 4C). By day 15 the HRI response in the tumor increased resulting in a significant difference between the two, with values of 2.3% and 1.1%. The HRI values in the tumor continued to increase reaching 3.2% on day 22 while the CLS HRI-values decreased to some extent to 0.6%. When examining the RTC graphs (fifth row), the increase in vessel reactivity over time is also demonstrated.

### Tumor response to B20

MRI-based assessment of glioblastoma tumor progression in this animal model demonstrated exponential tumor volume growth kinetics (Fig. 5A). An exponential model was fitted to each tumor and these curves were averaged and are presented in Fig. 5B. There was only a small delay in the growth rate of the B20-treated tumors. Yet, this difference was significant until day 20 in which the gap between the groups began to shrink. No significant difference was found, for the diffusion values, between the B20-treated animals and the control group, with the control group having slightly higher values (Fig. 5C). In both groups the tumor had higher ADC values than the contra lateral brain tissue.

Regarding tumor vasculature, heterogenic response within the brain-tumors was observed in HRI maps with higher values in the peripheral areas as opposed to tumors center (Fig. 4A and Fig. 5D). Similarly, CD31 staining confirmed the significantly higher vessel-count in the tumor periphery relative to tumor center (*p*<0.001; Fig. 2F and Fig. 5E). Unpredictably, HRI values of B20 treated tumors showed slightly higher values compared to control tumors (Fig. 5D), yet this difference was insignificant. In contrast, when analyzing the corresponding CD31 stained histological specimens, the B20 treated tumors showed significantly lower number of blood vessels (Fig. 5E).

Additionally, tumors of B20 treated mice showed significantly higher proliferation rate (Fig. 5F) compared to control tumors (18.5±4.3 compared to 6.2±5 respectively; p<0.0001). Moreover, B20 treated tumors also demonstrated significantly lower number of apoptotic cells (Fig. 5F) compared to control tumors (2.3±1.3 compared to 6±3 respectively; p<0.0001).

## Discussion

The major role of angiogenesis in the malignancy and prognosis of cancer on the one hand and as a treatment target on the other, necessitate the development of imaging techniques focusing on the vascular system. This is obviously true for human patients but is also essential in animal models due to their key function in the investigation of the underlying mechanisms and in the development of new drugs and novel cancer therapies. In the current study, HRI- a method based on changes in the BOLD-MRI signal caused by hyperoxia, was evaluated in a mouse model of glioblastoma treated with anti-angiogenic therapy. By using HRI the brain and tumor vasculature hemodynamic response was visualized and further characterized.

The combination of several anatomical and functional MRI protocols enabled improved glioblastoma progression and treatment response monitoring capabilities. Tumor borders were best distinguished on the true-FISP anatomical images. The tumor appeared brighter than the surrounding normal brain tissue and tumor volume was easily calculated. ADC values are increasingly utilized in the evaluation of patients with brain tumors, as these quantitative measures have been previously associated with increased cell density and disruption of normal tissue architecture [29], [30]. In the described mouse glioblastoma model the increased ADC values was almost constant throughout the entire tumor progression period. In addition, bright regions on FISP images parallel to regions with higher ADC values were noticed around the borders of some of the tumors suggesting areas of less cellularity and high water content as later confirmed by the H&E stained histological slides. Progressed tumor angiogenesis was clearly demonstrated using HRI and further confirmed by histological evaluation. Moreover, a steady and noticeable rise in the average HRI signal was seen during tumor progression, suggesting the use of HRI value as a biomarker for tumor vascularity and perfusion. Furthermore, a significant difference between the tumor border and center was measured using HRI which was later confirmed on CD31 immunostaining.

Angiogenesis inhibitors have been FDA approved leading to the routine use of the anti-VEGF antibody- bevacizumab in recurrent glioblastoma, conveying substantial improvements in progression-free survival and quality of life. Despite these encouraging beneficial effects, treated patients fail to demonstrate significantly improved overall survival [31]. In our mouse model, when comparing the B20 treated mice with the control group we found that while the CD31 based blood vessel count was significantly lower for the B20 treated group, no significant difference was found in the HRI analysis. This finding goes along with the current notion that while anti-VEGF antibodies such as bevacizumab reduce the number of blood vessels they also ‘normalize’ the tumor vasculature leading to improved blood flow [1]. Therefore, a smaller number of blood vessels could produce the same level of tissue perfusion. Although the B20 treatment slightly reduced tumor vascularity and delayed tumor progression, yet by day twenty the B20-treated mice closed the gap. Moreover, fewer apoptotic cells and higher proliferation index were detected in the B20-treated tumors compared to control-treated tumors. In previous work we similarly noticed reduced apoptosis in colorectal liver metastases treated with B20 [17]. Moreover, in the clinic, bevacizumab has been approved for the treatment of recurrent glioblastoma in combination with chemotherapy, while in our experiment B20 was given alone. These facts may elucidate the modest therapeutic effect of B20.

A main limitation in this model is the time scale. Human glioblastoma typically progresses over several months. The rodent model used in this study evolves over significantly shorter periods. This led to several discrepancies between the human and animal situations. In patients with glioblastoma we previously showed existence of vascular maturation by using HRI combined with hypercapnia [23]. In contrast in the mouse model, tumor vessels were not covered with vascular smooth muscle cells as assessed using α-SMA immunohistochemistry. An additional limitation is that only one cell line of glioblastoma was used and therefore results regarding the increased ADC and HRI values may be specific to this model. In addition, while GL26 cells produce very aggressive brain tumors in mice, these tumors are slightly different from those in humans as they are less infiltrative.

## Conclusions

This study presented the combination of several MRI protocols in the follow up and evaluation of a mouse model of glioblastoma treated with anti-angiogenic therapy. Due to the diversity of the different MRI protocols more comprehensive information is achieved when numerous protocols are used concomitantly. Special emphasis was given to assess functionality and the hemodynamic response of the brain vasculature. HRI provides a very easy, safe and contrast agent free method for monitoring the brain hemodynamic response. Since there is no contrast agent, it can be performed several times without any safety issues. Moreover, it is not based on the blood brain barrier breakdown, which is affected during anti-angiogenic-therapies.
